# Perinatal and neonatal outcomes of pregnancies after early rescue intracytoplasmic sperm injection in women with primary infertility compared with conventional intracytoplasmic sperm injection: a retrospective 6-year study

**DOI:** 10.1186/s12884-020-03155-9

**Published:** 2020-08-12

**Authors:** Feng Xiong, Qing Sun, Guangui Li, Zhihong Yao, Peilin Chen, Caiyun Wan, Huixian Zhong, Yong Zeng

**Affiliations:** Shenzhen Key Laboratory of Reproductive Immunology for Peri-implantation, Shenzhen Zhongshan Institute for Reproduction and Genetics, Fertility Center, Shenzhen Zhongshan Urology Hospital, Guangdong 518045 Shenzhen, People’s Republic of China

**Keywords:** Rescue ICSI, Fertilization failure, Primary infertility, Perinatal outcome, Neonatal outcome

## Abstract

**Background:**

Early rescue intracytoplasmic sperm injection (ICSI) has been used in clinic as appropriate currently. While the outcomes of children born after this method were not well assessed. The purpose of this study was to evaluate the effect of early rescue ICSI on women with primary infertility.

**Methods:**

Fresh embryo transfer cycles after rescue (*n* = 214) and conventional (*n* = 546) ICSI were retrospectively evaluated from women with primary infertility who underwent their first assisted reproductive technology cycles at our center in 2012–2017. The conventional ICSI group was subdivided into ICSI-1 (semen suitable for *in vitro* fertilization, IVF) and ICSI-2 (poor semen quality) to minimize bias from differences in semen quality. Pregnancy, delivery and neonatal outcomes were compared between groups.

**Results:**

There was a higher rate of polyspermy and a lower rate of top-quality embryos (TQE) on day 3 for oocytes subject to rescue ICSI compared with conventional ICSI. This reduced the total number of TQE and the number of TQE transferred in the rescue ICSI group. There was no significant difference between groups in clinical pregnancy, ongoing pregnancy, early miscarriage and live birth. For pregnant women, gestational age, route of delivery, risk of preterm birth and gestational diabetes mellitus were also comparable. Neonatal outcomes including sex ratio, birth weight, neonatal intensive care unit admission and birth defects were also similar after rescue and conventional ICSI. Moreover, no differences were observed with the different ICSI subgroups.

**Conclusions:**

For women with primary infertility who have a high risk of IVF fertilization failure (FF), rescue ICSI provides a safe and efficient alternative to minimize FF after initial IVF, but results in fewer TQE on day 3.

## Background

Infertility affects more than 186 million people worldwide, impacting on 8–12% of couples of reproductive age [1], and with a prevalence that increases annually. Infertility is divided into primary and secondary infertility depending on whether or not there has been a prior pregnancy. A high prevalence of primary infertility was reported in women of reproductive age [2]. *In vitro* fertilization (IVF) and intracytoplasmic sperm injection (ICSI) are currently the two most efficient techniques to help infertile women get pregnant. ICSI was developed more recently than IVF and is effective for most types of infertility including those that IVF cannot assist with. However, ICSI was only introduced in 1992 [3]. Therefore, it remains unclear whether it should be performed in all types of infertility because of potential safety concerns including lack of knowledge about the long-term effects on the offspring [4, 5]. The ICSI procedure is very different from the natural fertilization process *in vivo.* It entails mechanical removal of cumulus cells and subsequent invasive single sperm microinjection bypassing the zona pellucida, oolemma and the cytoplasmic organelle. Further, several studies have reported a significant increase in *de novo* sex and autosomal chromosome anomalies in the children derived from ICSI [6]. ICSI is also more expensive for patients since micromanipulation is more time consuming and requires greater technical skill [7]. Therefore, to optimize patient benefit, some IVF laboratories restrict ICSI mainly for when there is an extremely poor sperm sample or IVF has failed [8]. Thus, the more conservative strategy may be to use IVF as the first choice [9]. However, 4–20% of IVF cycles are associated with total fertilization failure (tFF) [10], adding an emotional and financial burden to an already stressful and expensive treatment plan. As a consequence, rescue ICSI has emerged to reduce the risk of tFF in the current IVF cycle [11].

Rescue ICSI was first introduced in 1993 for re-insemination on the second day (‘late rescue ICSI’) after IVF failure [12]. However, later attempts on 1-day-old oocytes yield poor results, probably due to oocyte aging [13]. Therefore, it was improved to perform earlier at 4–6 h after insemination (‘early rescue ICSI’) to avoid the decrease of oocyte quality, because a second polar body (PB) is released in 80% of fertilized oocytes by 4 h and in 90% of fertilized oocytes by 6 h [14, 15]. The oocytes were denuded after 4-hour co-incubation with spermatozoa and the number of PBs was checked. The unfertilized oocyte was identified by the presence of one PB and rescued with ICSI. It was reported in many studies that early rescue ICSI was superior to the former late approach [11]. In addition to effectively overcoming tFF, early rescue ICSI can save oocytes from a potentially detrimental environment contaminated by metabolic sperm degradation products by terminating sperm and oocyte incubation for the polar body (PB) early check [11]. Nevertheless, the outcomes of children born after early rescue ICSI are not well evaluated, mostly because patients who deliver after transferring embryos derived from rescue ICSI are challenging to recruit. One recent study reported on the safety and efficacy of early rescue ICSI. However, it was not well-designed because no inclusion or exclusion criteria were applied for included cycles [15].

Women with primary infertility in their first IVF cycle are probably most suited to short time insemination combined with early rescue ICSI when total or partial FF happened. This is because there is no indication that the oocytes can fuse with the sperm implied by previous fertilization. Our center has performed early PB check IVF and early rescue ICSI if necessary for all women with primary infertility in their first IVF cycle since 2012. We wanted to further evaluate the safety and efficacy of early rescue ICSI in clinical practice. Therefore, the purpose of this study was to retrospectively analyze the perinatal and neonatal outcomes of offspring following fresh cleavage embryo transfer (ET) after early rescue ICSI compared with conventional ICSI and ejaculated sperm.

## Methods

### Patients

**Fig. 1:**
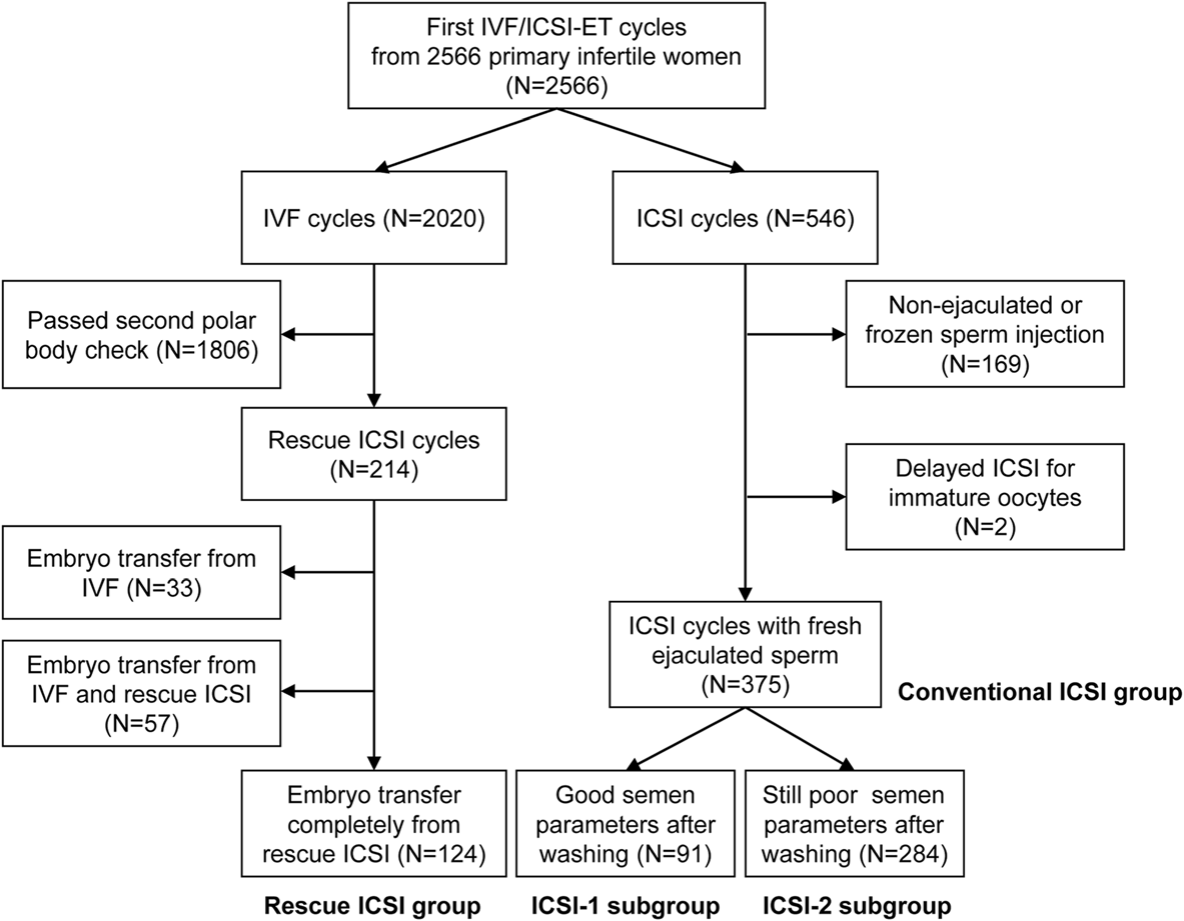
Cycles included in this study

A flowchart of patient recruitment is shown in Fig. 1. From January 2012 to December 2017, the first IVF/ICSI-ET cycle of 2566 women with primary infertility was evaluated retrospectively in a single fertility center. The oocytes from 2020 of these patients were checked for the presence of a second PB at 6 h after the initial IVF, with early rescue ICSI performed in 214 patients. This subset of individuals was recruited into the rescue ICSI group in the present study. For comparison, a total of 546 patients were recommended for initial treatment with ICSI because of poor semen parameters that were noted before oocyte retrieval. These patients were recruited into the conventional ICSI group. All cycles with at least one transferred embryo derived from IVF were excluded from the rescue ICSI group, whereas cycles that used non-ejaculated or frozen sperm and delayed ICSI cycles were excluded from the conventional ICSI group. Patients in the conventional ICSI group were subdivided into ICSI-1 and ICSI-2 groups according to semen parameters after washing on the day of ovum pick-up (OPU). The patients with semen parameters after washing beyond the conventional ICSI criteria outlined below were included in the ICSI-1 subgroup whereas those with semen quality after washing within the following ICSI criteria were included in ICSI-2.

### Conventional ICSI criteria

The decision to offer conventional ICSI was made by clinicians according to the semen parameters tested for the initial evaluation of infertility as an outpatient. If sperm concentration, motility or morphology met any one of the following conditions, ICSI was performed directly to minimize the risk of fertilization failure; otherwise, early PB check IVF is considered first and rescue ICSI was conducted if necessary:

a Initial spermatozoa concentration < 2 × 10^6^/mL;
b Percentage of progressive motile spermatozoa < 25%, or percentage of total motile spermatozoa < 40% when initial spermatozoa concentration was 2–20 × 10^6^/mL;
c Percentage of total motile spermatozoa < 5% when initial spermatozoa concentration > 20 × 10^6^/mL;
d Percentage of normal spermatozoa < 1%.

The aforementioned conventional ICSI criteria were self-ordained by our center.

### Laboratory procedures

The long down-regulation or antagonist protocol was used in controlled ovarian stimulation. OPU was performed under transvaginal ultrasound guidance at 36 h after human chorionic gonadotropin (HCG) administration. Simultaneously, ejaculated spermatozoa were washed using the density gradient centrifugation method. Insemination or ICSI was performed 40–42 h after HCG injection. Quinn’s Advantage embryo culture system (CooperSurgical, Trumbull, USA) was used for embryo culture *in vitro* as per the manufacturer’s instructions. The quality of cleavage embryos was evaluated on day 3 according to the morphological criteria described previously [16], and up to three of the best embryos from each patient were selected for transfer under ultrasound guidance that day.

### Early PB check procedure and rescue ICSI criteria

Early PB check was performed for all women with primary infertility in their first IVF cycle at our center. For cumulus-oocyte-complexes from these patients, granular cells were removed by a finely drawn glass pipette after 4 h of oocyte and spermatozoa incubation and the second PB was observed under the inverted microscope (400×). If fewer than 30% of the mature oocytes released a second PB, culture continued for all the oocytes for more than two hours and re-checked for the second PB exposure. Rescue ICSI was performed immediately on the oocytes with only one PB when there were still fewer than 50% of the mature oocytes exposing the second PB. The procedure for rescue ICSI was the same as for conventional ICSI. Briefly, The oocyte was positioned in the dish with the first PB at 6 o’clock and sperm injection was performed at 3 o’clock. Narishige manipulation system (Narishige, Tokyo, Japan) and micropipette with 5 µm inner diameter (The Pipette Company, Thebarton, South Australia ) were used in the procedure. Notably, It was difficult to identify the number of PBs when they are fragmented, then rescue ICSI can be considered if the status of PBs did not change in the re-check two hours later, according to the previous study [17].

### Outcomes evaluation

Pregnancy, delivery outcomes and neonatal outcomes were analyzed in both the rescue and conventional ICSI groups. The outcome parameters were evaluated according to the definitions in Table 1.

**Table 1:** Definition of outcome parameters

Parameter	Definition
Clinical pregnancy	Observation of at least one intrauterine gestational sac on ultrasound one month after embryo transfer
Implantation rate	Number of gestational sacs per number of embryos transferred one month after embryo transfer
Ectopic pregnancy	One or more gestational sacs outside the uterus confirmed by sonography or laparoscopy
Early miscarriage	Complete loss of the fetus before three months’ gestation
Multiple pregnancy	Two or more gestational sacs or positive heartbeats confirmed by transvaginal sonography one month after embryo transfer
Ongoing pregnancy	Pregnancy completed to three months or more of gestation
Live birth	Delivery of one or more infants with any signs of life.
Preterm delivery	Delivery at < 37 completed weeks’ gestation
Very preterm delivery	Delivery at < 32 completed weeks’ gestation
Low birth weight	Weight of newborn < 2500 g at birth
Very low birth weight	Weight of newborn < 1500 g at birth
Small for gestational age	Weight of newborn < 10th percentile
Admission to NICU	Admittance of the newborn to NICU after birth

*NICU* neonatal intensive care unit

#### Statistical analysis

SPSS version 24 (IBM, USA) was used for all statistical analyses. Quantitative variables were analyzed by Student’s *t*-test and one-way analysis of variance (ANOVA) to compare the differences, and the least square difference (LSD) test for post-hoc comparisons. Categorical variables were analyzed using the Chi-squared test.

Multivariate logistic regression was performed to evaluate the relationship between rescue ICSI or conventional ICSI and clinical outcomes by adjusting for potential confounding factors. For the pregnancy outcomes, adjustments were made for maternal age, body mass index (BMI), number of top-quality embryos (TQE) transferred, and maternal infertility diagnosis. For delivery and neonatal outcomes, adjustments were made for maternal age, BMI, number of TQE transferred, maternal infertility diagnosis, single or multiple pregnancy, and singleton or twin delivery. For twin deliveries, the generalized estimating equation method was used to evaluate differences in the neonatal outcomes combined with multivariate logistic regression [18]. The crude and adjusted odds ratios were calculated along with the associated 95% confidence intervals.

## Results

Baseline characteristics of the studied patients are shown in Table 2. There was no significant difference between the rescue and conventional ICSI groups, except for a higher BMI in the rescue ICSI group and variation in maternal infertility diagnosis between groups. Paternal age, infertility duration, follicle counts on HCG day and the number of oocytes retrieved were comparable. Focusing on the oocytes re-inseminated by rescue ICSI, there were similar normal fertilization and damaging rates, but a lower TQE rate on day 3 and a higher polyspermy rate compared with conventional ICSI. Moreover, the total number of TQE on day 3 in the rescue ICSI group was lower than in the conventional ICSI group. Therefore, a significantly decreased number of TQE able to be transferred was found in the rescue ICSI group. Nevertheless, the average number of embryos transferred and the endometrial thickness was similar between the two groups (Table 2).

**Table 2:** Baseline characteristic of patients in the rescue and conventional ICSI groups

	Rescue ICSI group	Conventional ICSI group		
		Total	ICSI-1 subgroup	ICSI-2 subgroup
Patients	124	375	91	284
Maternal age (years)	31.02 ± 3.50^ab^	30.63 ± 3.85	31.32 ± 3.98^a^	30.42 ± 3.79^b^
Paternal age (years)	34.12 ± 5.86	33.65 ± 5.43	33.05 ± 4.68	33.85 ± 5.65
BMI (kg/m^2^)	21.77 ± 3.00^a^	21.08 ± 2.93^*^	21.07 ± 3.48^ab^	21.08 ± 2.75^b^
Infertility duration (years)	4.42 ± 3.03	3.93 ± 2.72	3.86 ± 2.71	3.95 ± 2.73
Maternal infertility diagnosis, n (%)				
Tubal factor	35 (28.23)	106 (28.27)	28 (30.77)	78 (27.46)
Ovulation dysfunction	7 (5.65)	25 (6.67)	8 (8.79)	17 (5.99)
Endometriosis	19 (15.32) ^a^	19 (5.07)^*^	9 (9.89) ^a^	10 (3.52) ^b^
Unexplained infertility	63 (50.81)	225 (60.00)	46 (50.55)	179 (63.03)
Follicle counts on HCG day	9.90 ± 3.22	9.74 ± 3.15	9.45 ± 3.05	9.84 ± 3.18
No. of oocytes retrieved	12.82 ± 5.21	12.63 ± 5.59	12.47 ± 5.76	12.69 ± 5.55
No. of oocytes injected	8.92 ± 4.54^b^	10.63 ± 5.04^*^	10.12 ± 4.87^ab^	10.80 ± 5.09^a^
Embryo outcomes per injected oocyte, n (%)				
Normal fertilization rate	859/1106 (77.67)	3125/3987 (78.38)	733/921 (79.59)	2392/3066 (78.02)
Polyspermy rate	27/1106 (2.44)	56/3987 (1.40)^*^	13/921 (1.41)	43/3066 (1.40)
TQE rate	473/1106 (42.77)^c^	1933/3987 (48.48)^*^	479/921 (52.01)^a^	1454/3066 (47.42)^b^
Damage oocyte rate	60/1106 (5.42)	173/3987 (4.34)	50/921 (5.43)	123/3066 (4.01)
Total no. of TQE	4.02 ± 3.42^b^	5.15 ± 3.62^*^	5.26 ± 3.74^a^	5.12 ± 3.60^a^
No. of embryo transferred	2.09 ± 0.38	2.14 ± 0.37	2.15 ± 0.39	2.13 ± 0.37
No. of TQE transferred	1.75 ± 0.73^b^	1.96 ± 0.63^*^	1.97 ± 0.66^a^	1.95 ± 0.63^a^
Endometrial thickness (mm)	11.67 ± 2.16	11.87 ± 2.56	11.62 ± 2.52	11.95 ± 2.57

^*^p value < 0.05 compared to rescue ICSI group

^a,b,c,d^ Values with different superscript letters indicate significant differences between different subgroups. *P* < 0.05 indicates significance in multiple comparisons of quantitative data; *P* < 0.017 indicates significance in multiple comparisons of categorical data

*ICSI* intracytoplasmic sperm injection, *BMI* body mass index, *HCG* human chorionic gonadotropin, *TQE* top-quality embryo

Analysis of the subdivided conventional ICSI group revealed that there was a younger maternal age in the ICSI-2 subgroup compared to both ICSI-1 and rescue ICSI groups, and the BMI of women in the ICSI-2 subgroup was significantly lower than in the rescue ICSI group. Similarly, the rate of TQE developed from the oocytes that had undergone rescue ICSI and the average number of TQE on day 3 were lower than in both the ICSI-1 and ICSI-2 subgroups. Further, there was a lower number of TQE transferred in the rescue ICSI group compared with both the ICSI-1 and ICSI-2 subgroups (Table 2).

The pregnancy, delivery and neonatal outcomes of pregnant women in the rescue ICSI group were analyzed and compared with those of the conventional ICSI group. The results are shown in Table 3. The rate of clinical pregnancy, ectopic pregnancy, early miscarriage, ongoing pregnancy and live birth were similar between the two groups. The implantation rate of embryos derived from rescue ICSI was not significantly decreased compared with those from conventional ICSI. In terms of delivery outcomes, singleton and twin delivery rates were comparable between rescue and conventional ICSI groups. There was no significant difference between groups for gestational days, preterm birth, very preterm birth, route of delivery, or gestational diabetes mellitus. A total of 84 babies were delivered in the rescue ICSI group and 274 babies were delivered in the conventional ICSI group. In terms of neonatal outcomes, the sex ratio, birth weight, and rates of low birth weight (LBW), very low birth weight (VLBW), small for gestation (SGA), admission to neonatal intensive care unit (NICU), and birth defects were not significantly different between groups. Further evaluation of the outcomes mentioned above for comparison between the ICSI-1 or ICSI-2 subgroups and the rescue ICSI group did not reveal a significant difference for the conventional ICSI subgroups.

**Table 3:** Pregnancy, delivery and neonatal outcomes of pregnant patients in the rescue ICSI and conventional ICSI groups

	Rescue ICSI group	Conventional ICSI group		
		Total	ICSI-1 subgroup	ICSI-2 subgroup
**Pregnancy outcomes**				
Clinical pregnancy, n (%)	68 (54.84)	232 (61.87)	52 (57.14)	180 (63.38)
Single pregnancy, n (%)	33 (48.53)	127 (54.74)	26 (50.00)	101 (56.11)
Twin pregnancy, n (%)	34 (50.00)	99 (42.67)	25 (48.08)	74 (41.11)
Multiple pregnancy, n (%)	1 (1.47)	6 (2.59)	1 (1.92)	5 (2.78)
Ectopic pregnancy per transfer, n (%)	2 (1.61)	3 (0.80)	1 (1.10)	2 (0.70)
Multifetal pregnancy reduction, n (%)	2 (2.94)	9 (3.88)	4 (7.69)	5 (2.78)
Implantation, n (%)	102/259 (39.38)	343/801 (42.82)	80/196 (40.82)	263/605 (43.47)
Early miscarriage, n (%)	5 (7.35)	23 (9.91)	3 (5.77)	20 (11.11)
Ongoing pregnancy per transfer, n (%)	63 (50.81)	209 (55.73)	49 (53.85)	160 (56.34)
Live deliveries per transfer, n (%)^a^	62 (50.41)	200 (53.48)	47 (51.65)	153 (54.06)
**Delivery outcomes**				
Twins, n (%)	22 (35.48)	74 (37.00)	18 (38.30)	56 (36.60)
Singleton, n (%)	40 (64.52)	126 (63.00)	29 (61.70)	97 (63.40)
Gestational days	267.68 ± 15.39	266.86 ± 15.34	266.83 ± 16.87	266.86 ± 14.90
Preterm birth (< 37 wk), n (%)	14 (22.95)	44 (22.56)	10 (22.73)	34 (25.52)
Very preterm birth (< 32 wk), n (%)	1 (1.64)	4 (2.05)	2 (4.55)	2 (1.32)
Cesarean section, n (%)	41 (66.13)	141 (70.50)	30 (63.83)	111 (72.55)
Vaginal deliveries, n (%)	21 (33.87)	59 (29.50)	17 (36.17)	42 (27.45)
GDM, n (%)	8 (12.90)	22 (11.00)	5 (10.64)	17 (11.11)
**Neonatal outcomes**				
Live birth	84	274	65	209
Male neonates, n (%)	46 (54.76)	132 (48.18)	34 (52.31)	98 (46.89)
Female neonates, n (%)	38 (45.23)	142 (51.82)	31 (47.69)	111 (53.11)
Birth weight (g)	2792.62 ± 571.77	2742.82 ± 598.32	2688.95 ± 540.02	2759.57 ± 615.58
Male birth weight (g)	2794.57 ± 585.03	2803.58 ± 611.79	2649.76 ± 535.81	2856.94 ± 629.80
Female birth weight (g)	2790.26 ± 563.11	2686.34 ± 581.98	2731.94 ± 550.17	2673.60 ± 592.33
LBW (<2500 g), n (%)	23 (27.38)	84 (30.66)	18 (27.69)	66 (31.58)
VLBW (<1500 g), n (%)	1 (1.19)	5 (1.82)	1 (1.54)	4 (1.91)
SGA, n (%)	3 (3.57)	26 (9.49)	7 (10.77)	19 (9.09)
Admission to NICU, n (%)	11 (13.10)	38 (13.87)	11 (16.92)	27 (12.92)
Days at the NICU	10.09 ± 6.59	15.55 ± 13.47	19.91 ± 11.03	13.78 ± 14.15
Birth defects, n (%)	2 (2.38)	4 (1.46)	1 (1.54)	3 (1.44)

Comparisons were made between the rescue ICSI group and the conventional ICSI group, and also within the two subgroups, but no significant difference was found

*ICSI* intracytoplasmic sperm injection, *GDM* gestational diabetes mellitus, *LBW* low birth weight, *VLBW* very low birth weight, *SAG* small for gestation age, *NICU* neonatal intensive care unit

^a^One case of live birth outcome missing in rescue ICSI group and ICSI-2 subgroup, respectively

Furthermore, we found that rescue ICSI did not compromise the perinatal or neonatal outcomes analyzed in our study after adjusting for potential confounders. The rates of clinical pregnancy, ectopic pregnancy, ongoing pregnancy, early miscarriage, and live birth were comparable between groups after adjusting for maternal age, BMI, maternal infertility reasons, and number of TQE transferred (Table 4). Similarly, gestational age, route of delivery, occurrence of gestational diabetes mellitus, birth weight, and rate of birth defects were also similar in the two groups after adjusting for maternal age, BMI, maternal infertility reasons, number of TQE transferred, single or multiple pregnancy, occurrence of pregnancy reduction, and singleton or twin delivery (Table 5).

**Table 4:** Logistic regression analysis and adjusted odds ratios for pregnancy outcomes in the rescue ICSI group compared with the conventional ICSI group

	Crude		Adjusted	
	OR (95% CI)	*P* value	OR (95% CI)	*P* value
Clinical pregnancy	0.760 (0.502–1.150)	0.194	0.888 (0.568–1.389)	0.604
Ectopic pregnancy	2.033 (0.336–12.308)	0.440	2.201 (0.347–13.959)	0.402
Early miscarriage	0.721 (0.263–1.975)	0.525	0.594 (0.208–1.692)	0.329
Ongoing pregnancy	0.820 (0.546–1.232)	0.340	0.988 (0.638–1.530)	0.956
Live deliveries	0.884 (0.588–1.329)	0.554	1.074 (0.694–1.661)	0.749

List of variables used to adjust OR: maternal age, BMI, maternal infertility diagnosis, the number of top-quality embryo transferred

*ICSI* intracytoplasmic sperm injection, *OR* odds ratio, *CI* confidence interval, *BMI* body mass index

**Table 5:** Logistic regression analysis and adjusted odds ratios for delivery and neonatal outcomes in the rescue ICSI group compared with the conventional ICSI group

	Crude		Adjusted	
	OR (95% CI)	*P* value	OR (95% CI)	*P* value
Preterm birth	1.034 (0.522–2.047)	0.923	1.222 (0.551–2.712)	0.621
Very preterm birth	0.803 (0.088–7.323)	0.846	0.578 (0.051–6.544)	0.658
Cesarean sections	0.817 (0.445-1.500)	0.514	0.739 (0.355–1.536)	0.418
GDM	1.199 (0.505–2.846)	0.681	1.095 (0.431–2.783)	0.849
Male neonates	1.310 (0.794–2.160)	0.291	1.177 (0.692–2.002)	0.548
LBW	0.966 (0.508–1.837)	0.916	0.895 (0.429–1.866)	0.767
SGA	0.501 (0.138–1.820)	0.294	0.391 (0.102–1.504)	0.172
Admission to NICU	1.230 (0.519–2.913)	0.638	1.316 (0.537–3.224)	0.548

List of variables used to adjust OR: maternal age, BMI, maternal infertility diagnosis, number of top-quality embryo transferred, occurrence of multifetal pregnancy reduction, single or multiple pregnancy, and singleton or twin delivery

*ICSI* intracytoplasmic sperm injection, *OR* odds ratio, *CI* confidence interval, *GDM* gestational diabetes mellitus, *LBW* low birth weight, *SAG* small for gestation age, *NICU* neonatal intensive care unit, *BMI* body mass index

Neonatal outcomes in terms of very low birth weight (VLBW) and birth defect were not analyzed by the generalized estimating equations method because a quasi-complete separation existed in the limited available data and the maximum likelihood estimates did not exist

## Discussion

For insemination, oocytes can be fertilized after exposure to spermatozoa for 2–4 h. The earliest sign of successful fertilization that can be observed in the laboratory is release of the second PB. Previous research indicates that the second PB is released in 80% of fertilized oocytes by 4 h and in 90% of fertilized oocytes by 6 h [14]. Therefore, checking for the second PB at 4–6 h after initial insemination can efficiently evaluate the risk of tFF or near tFF. A novel theoretical approach is then to perform rescue ICSI in oocytes lacking a second PB. However, the clinical safety and efficacy of rescue ICSI have been poorly investigated to date. Beck-Fruchter et al. systematically reviewed five studies from 1992 to 2013 and concluded that rescue ICSI can result in the delivery of healthy babies. However, this was based on limited and incomplete data [19]. Recently, a long-term retrospective study reported similar clinical pregnancy rates and neonatal health with rescue ICSI compared with conventional IVF and ICSI [15]. Unfortunately, this study included “all comers” without defining any inclusion and exclusion criteria. Our study focused on evaluating the clinical outcomes of the first cycle of women with primary infertility who are probably most suitable for rescue ICSI after FF with initial IVF. The results showed no significant differences in pregnancy, delivery or neonatal outcomes after fresh cleavage-stage embryo transfer with rescue versus conventional ICSI, which further confirmed the previous findings with more homogeneous data [15]. Moreover, to minimize bias derived from semen parameters in present study, patients in the ICSI group were further divided into ICSI-1 and ICSI-2 subgroups according to semen quality tested on the oocyte retrieval day. Semen quality in the ICSI-1 subgroup was suitable for IVF, and clinical outcomes were found similar between the rescue ICSI group and the ICSI-1 subgroup. However, decreased TQE developed on day 3 in the rescue ICSI group, with a similar number of oocytes retrieved compared with the conventional ICSI group. The influence of different semen parameters between rescue ICSI and conventional ICSI group was not considered in previous study [15], although they probably affect the embryo development [20, 21] and chromosomal status [22].

Our current knowledge indicates that an oocyte in the fallopian tube after ovulation loses activity gradually if fertilization does not occur within 48 h. The process is similar *in vitro*, with an oocyte aging if it is not fertilized by spermatozoa and progressed to the cleavage stage [23]. Therefore, it is clear why earlier rescue ICSI was superior to re-insemination conducted at a later time. In consideration of the accuracy of fertilization evaluation based on the presence of a second PB, rescue ICSI was performed 6 h after initial insemination. This is probably the optimal time for rescue ICSI before oocyte aging but after release of the second PB [24]. In this way, the main risk of rescue ICSI, polyspermic fertilization [25], can be decreased. However, it must be mentioned that polyspermic fertilization cannot be avoided in rescue ICSI cycles even with very experienced embryologists. It is very important to balance the time-related risks of oocyte aging and polyspermic fertilization for rescue ICSI. This is probably the reason for the decreased number of TQE on day 3 in the rescue ICSI group compared with the conventional ICSI group, because both oocyte aging and polyspermic fertilization can result in poor embryo quality. However, the best morphological embryo was the priority to transfer in the lab and so the pregnancy, delivery and neonatal outcomes in the first ET cycle were not significantly compromised in this study. It was supposed that the cumulative pregnancy rate and cumulative delivery rate were probably reduced in rescue ICSI group owing to the decreased number of TQE on day 3.

Conventional ICSI was performed 3–4 h after OPU and rescue ICSI was performed 6–7 h post OPU in the study. The similar outcomes of rescue ICSI compared with conventional ICSI indicated a large range of time is available for successful performance of ICSI. Previous substantial studies have examined the optimal time for oocyte denuding and ICSI. While it remains controversial, it has been reported that a 2–6 h interval between oocyte retrieval and ICSI can improve oocyte maturation, fertilization, embryo quality and even pregnancy rate [26–28]. This optimal timing is probably associated with the spindle presence in the oocyte [29, 30]. Other studies have found no significant differences in reproductive outcomes across a wide time interval range between oocyte retrieval and ICSI [31]. A recent study in which time intervals between OPU and ICSI ranged from 1 h 25 min to 17 h 13 min revealed no significant difference in reproductive outcomes including biochemical, ongoing and live pregnancy rate [32]. In our protocols, rescue ICSI was performed 6 h after initial insemination and about 10 h after OPU. The similar pregnancy and live birth rate in our study were consistent with the findings above that the wide interval range between OPU and ICSI seems not to affect the pregnancy outcomes. Furthermore, we also found no significant difference in delivery and neonatal outcomes between rescue and conventional ICSI group, which indicate the efficacy and safety of a broad time range for oocyte injection. The associations between ICSI timing and delivery or neonatal outcomes were not investigated previously to our knowledge.

Fertilization is a complex process that successively includes sperm penetration, extrusion of the second PB, oocyte activation, decondensation of both nuclei, and chromosome cytoplasmic migration of the pronuclear. A fault with any of the individual steps could cause FF, although it has been reported that most FFs in IVF are due to failure of sperm to penetrate into the oocyte [19, 33], and this is probably the main reason for absence of a second PB in oocytes retrieved from women with primary infertility. Early rescue ICSI solves sperm penetration issues by injecting sperm into the oocyte mechanically before oocyte aging. Thus, embryo quality and clinical outcomes are not comprised in most cases.

Limitations of this study include its retrospective nature and limited sample size due to the low frequency of rescue ICSI occurrence. We focus on the women with primary infertility, who are probably most suitable for early second PB check. Because previous pregnancy history of secondary infertile female indicates the ability of sperm-egg binding, IVF can be directly performed. However, most of the women with primary infertility are young and normal responders, which resulted in the outcomes of advanced age women and poor responders were not investigated in present study. Although we divided the ICSI group into two subgroups according to semen parameters, it was still difficult to avoid possible bias from unbalanced semen quality. Therefore, prospective multicenter cohort studies are needed to provide more evidence for the efficacy of rescue ICSI.

## Conclusions

In conclusion, we investigated the effects of early rescue ICSI in women with primary infertility who are at a high risk of IVF FF. Our findings showed a higher rate of polyspermy and a lower rate of TQE on day 3 for the oocytes that underwent rescue ICSI. This resulted in a decrease in the total amount of TQE embryos and, subsequently, a reduction in the number of transferred TQE in the rescue ICSI group. However, pregnancy, delivery and neonatal health outcomes after fresh transfer of cleavage embryos derived from early rescue ICSI were not compromised. In fact, our findings suggest that it is best to perform ICSI directly on patients suffering from failure extrusion of the second PB. However, it is difficult to predict outcomes if IVF is not performed first. Our study shows that rescue ICSI is a safe and efficient choice of fertilization method for women with primary infertility in their first conception cycle *in vitro*. It potentially offers great value in countries or IVF centers that use ICSI conservatively for reasons such as safety concerns or economic burden.

## Data Availability

The original data of this study are available from the corresponding author on reasonable request.

## Abbreviations

BMI: Body mass index
CI: Confidence interval
ET: Embryo transfer
FF: Fertilization failure
GDM: Gestational diabetes mellitus
HCG: Human chorionic gonadotropin
ICSI: Intracytoplasmic sperm injection
IVF: In vitro fertilization
LBW: Low birth weight
NICU: Neonatal intensive care unit
OPU: Ovum pick-up
OR: Odds ratio
PB: Polar body
SAG: Small for gestation age
TQE: Top-quality embryo
VLBW: Very low birth weight

## References

[1] Xiong F, Sun Q, Li G, Yao Z, Chen P, Wan C (2020). Association between the number of morphological top-quality blastocysts developed and live births after single blastocyst transfer in the first fresh or frozen IVF/ICSI cycle. Reprod Biomed Online.

[2] Zhou Z, Zheng D, Wu H, Li R, Xu S, Kang Y (2018). Epidemiology of infertility in China: a population-based study. BJOG.

[3] Palermo G, Joris H, Devroey P, Van Steirteghem AC (1992). Pregnancies after intracytoplasmic injection of single spermatozoon into an oocyte. Lancet.

[4] Dang VQ, Vuong LN, Ho TM, Ha AN, Nguyen QN, Truong BT (2019). The effectiveness of ICSI versus conventional IVF in couples with non-male factor infertility: study protocol for a randomised controlled trial. Hum Reprod Open.

[5] Wolff MV, Haaf T. In Vitro Fertilization Technology and Child Health. Dtsch Arztebl Int. 2020;117(3):23–30. DOI:10.3238/arztebl.2020.0023. 10.3238/arztebl.2020.0023 PMC7026576 32031509

[6] Simopoulou M, Giannelou P, Bakas P, Gkoles L, Kalampokas T, Pantos K (2016). Making ICSI Safer and More Effective: A Review of the Human Oocyte and ICSI Practice. In Vivo.

[7] Palermo GD, Neri QV, Rosenwaks Z (2015). To ICSI or Not to ICSI. Semin Reprod Med.

[8] Palermo GD, Neri QV, Takeuchi T, Rosenwaks Z (2009). ICSI: where we have been and where we are going. Semin Reprod Med.

[9] Drakopoulos P, Garcia-Velasco J, Bosch E, Blockeel C, de Vos M, Santos-Ribeiro S (2019). ICSI does not offer any benefit over conventional IVF across different ovarian response categories in non-male factor infertility: a European multicenter analysis. J Assist Reprod Genet.

[10] Arpit S, Ji Y (2017). Is Rescue Intracytoplasmic Sperm Injection, an Option for Fertilization Failure? A Systematic Review. Science Letters.

[11] He Y, Liu H, Zheng H, Li L, Fu X, Liu J (2018). Effect of early cumulus cells removal and early rescue ICSI on pregnancy outcomes in high-risk patients of fertilization failure. Gynecol Endocrinol.

[12] Nagy ZP, Joris H, Liu J, Staessen C, Devroey P, Van Steirteghem AC (1993). Intracytoplasmic single sperm injection of 1-day-old unfertilized human oocytes. Hum Reprod.

[13] Sjogren A, Lundin K, Hamberger L (1995). Intracytoplasmic sperm injection of 1 day old oocytes after fertilization failure. Hum Reprod.

[14] Chen C, Kattera S (2003). Rescue ICSI of oocytes that failed to extrude the second polar body 6 h post-insemination in conventional IVF. Hum Reprod.

[15] Huang B, Qian K, Li Z, Yue J, Yang W, Zhu G (2015). Neonatal outcomes after early rescue intracytoplasmic sperm injection: an analysis of a 5-year period. Fertil Steril.

[16] Wan CY, Song C, Diao LH, Li GG, Bao ZJ, Hu XD (2014). Laser-assisted hatching improves clinical outcomes of vitrified-warmed blastocysts developed from low-grade cleavage-stage embryos: a prospective randomized study. Reprod Biomed Online.

[17] Liu W, Liu J, Zhang X, Han W, Xiong S, Huang G (2014). Short co-incubation of gametes combined with early rescue ICSI: an optimal strategy for complete fertilization failure after IVF. Hum Fertil (Camb).

[18] Cobo A, Serra V, Garrido N, Olmo I, Pellicer A, Remohi J (2014). Obstetric and perinatal outcome of babies born from vitrified oocytes. Fertil Steril.

[19] Beck-Fruchter R, Lavee M, Weiss A, Geslevich Y, Shalev E (2014). Rescue intracytoplasmic sperm injection: a systematic review. Fertil Steril.

[20] Mangoli E, Khalili MA, Talebi AR, Ghasemi-Esmailabad S, Hosseini A (2018). Is there any correlation between sperm parameters and chromatin quality with embryo morphokinetics in patients with male infertility?. Andrologia.

[21] Tarozzi N, Nadalini M, Lagalla C, Coticchio G, Zaca C, Borini A (2019). Male factor infertility impacts the rate of mosaic blastocysts in cycles of preimplantation genetic testing for aneuploidy. J Assist Reprod Genet.

[22] Magli MC, Gianaroli L, Ferraretti AP, Gordts S, Fredericks V, Crippa A (2009). Paternal contribution to aneuploidy in preimplantation embryos. Reprod Biomed Online.

[23] Miao YL, Kikuchi K, Sun QY, Schatten H (2009). Oocyte aging: cellular and molecular changes, developmental potential and reversal possibility. Hum Reprod Update.

[24] Zhu LX, Renx L, Li W, Juan H, Li YF, Zhang HW (2011). Rescue ICSI: choose the optimal rescue window before oocyte aging. J Reprod Contraception..

[25] Esfandiari N, Claessens EA, Burjaq H, Gotlieb L, Casper RF (2008). Ongoing twin pregnancy after rescue intracytoplasmic sperm injection of unfertilized abnormal oocytes. Fertil Steril..

[26] Ho JY, Chen MJ, Yi YC, Guu HF, Ho ES (2003). The effect of preincubation period of oocytes on nuclear maturity, fertilization rate, embryo quality, and pregnancy outcome in IVF and ICSI. J Assist Reprod Genet.

[27] Falcone P, Gambera L, Pisoni M, Lofiego V, De Leo V, Mencaglia L (2008). Correlation between oocyte preincubation time and pregnancy rate after intracytoplasmic sperm injection. Gynecol Endocrinol.

[28] Patrat C, Kaffel A, Delaroche L, Guibert J, Jouannet P, Epelboin S (2012). Optimal timing for oocyte denudation and intracytoplasmic sperm injection. Obstet Gynecol Int.

[29] Cohen Y, Malcov M, Schwartz T, Mey-Raz N, Carmon A, Cohen T (2004). Spindle imaging: a new marker for optimal timing of ICSI?. Hum Reprod.

[30] Pujol A, Garcia D, Obradors A, Rodriguez A, Vassena R (2018). Is there a relation between the time to ICSI and the reproductive outcomes?. Hum Reprod.

[31] Maggiulli R, Cimadomo D, Fabozzi G, Papini L, Dovere L, Ubaldi FM (2020). The effect of ICSI-related procedural timings and operators on the outcome. Hum Reprod.

[32] Barcena P, Rodriguez M, Obradors A, Vernaeve V, Vassena R (2016). Should we worry about the clock? Relationship between time to ICSI and reproductive outcomes in cycles with fresh and vitrified oocytes. Hum Reprod.

[33] Mahutte NG, Arici A (2003). Failed fertilization: is it predictable?. Curr Opin Obstet Gynecol.

